# Predicting links between tumor samples and genes using 2-Layered graph based diffusion approach

**DOI:** 10.1186/s12859-019-3056-2

**Published:** 2019-09-09

**Authors:** Mohan Timilsina, Haixuan Yang, Ratnesh Sahay, Dietrich Rebholz-Schuhmann

**Affiliations:** 1 Insight Centre for Data Analytics, National University of Ireland Galway, Galway, Ireland; 2 School of Mathematics Statistics and Applied Mathematics, National University of Ireland Galway, Galway, Ireland

**Keywords:** Graph, Heat, Diffusion, Prediction, Tumor, Genes, Interaction

## Abstract

**Background:**

Determining the association between tumor sample and the gene is demanding because it requires a high cost for conducting genetic experiments. Thus, the discovered association between tumor sample and gene further requires clinical verification and validation. This entire mechanism is time-consuming and expensive. Due to this issue, predicting the association between tumor samples and genes remain a challenge in biomedicine.

**Results:**

Here we present, a computational model based on a heat diffusion algorithm which can predict the association between tumor samples and genes. We proposed a 2-layered graph. In the first layer, we constructed a graph of tumor samples and genes where these two types of nodes are connected by “hasGene” relationship. In the second layer, the gene nodes are connected by “interaction” relationship. We applied the heat diffusion algorithms in nine different variants of genetic interaction networks extracted from STRING and BioGRID database. The heat diffusion algorithm predicted the links between tumor samples and genes with mean AUC-ROC score of 0.84. This score is obtained by using weighted genetic interactions of fusion or co-occurrence channels from the STRING database. For the unweighted genetic interaction from the BioGRID database, the algorithms predict the links with an AUC-ROC score of 0.74.

**Conclusions:**

We demonstrate that the gene-gene interaction scores could improve the predictive power of the heat diffusion model to predict the links between tumor samples and genes. We showed the efficient runtime of the heat diffusion algorithm in various genetic interaction network. We statistically validated our prediction quality of the links between tumor samples and genes.

**Electronic supplementary material:**

The online version of this article (10.1186/s12859-019-3056-2) contains supplementary material, which is available to authorized users.

## Background

Traditionally, the linkage and mapping between genes and diseases are well researched using genome-scale-sequence-based associations studies [1]. The studies of the disease causal genes are essential, but because of their time-consuming approach to experimental validation, these methods are considered expensive. The sequencing of the genes and associations studies performs better for carefully selected functional gene candidates. This process is complicated and demands specialized knowledge [2]. Phenotypically similar diseases are often caused by functionally related genes [3], which strongly motivates the use of graph analytics and network science to study the functional relevance of the genes.

The genetic variation accounts for a proportion of susceptibility to common diseases such as diabetes, cardiovascular disease, and tumor. In the context of the tumor, DNA methylation is one of the early diagnostic markers of cancer. The differential DNA methylation status of each gene in each tumor can be verified and successfully accomplished using gene expression data in the laboratory settings. However, identifying the methylated DNA genes can only be obtained through laborious and tedious experiments. Consequently, DNA methylation-based (DNAm) studies in tumors are complicated further by disease heterogeneity [4].

In this study, we have used DNA methylation data. The DNA methylation is most intensely studied in epigenetic modifications in mammals and has important roles in studying tumorigenesis [5, 6]. A causal relationship between inflammation and cancer has long been accepted in multiple tumor types, supported by the evidence that the methylated gene observed in early dysplastic lesion [7]. The DNA methylation represents an early and crucial step in the gene-regulation pathway by which normal tissue experiences neoplastic transformation for the development of cancer. Further, a judgment of the methylation profiles within neoplastic tissues help the diagnosis of the disease, predicting the clinical behavior, and designing specific treatment plans for individual patients.

Every tumor samples are extracted from the individual patients. Thus conducting biological experiment to identify methylated cancer genes for each tumor samples is expensive and cumbersome. Therefore, computational methods can be a complementary approach [8, 9] because such models are faster and cheaper to perform than biological assessment.

There are large number of studies on tumor-type identification in a laboratory setting. Tumor tissues(biopsy) remain the main method for analyzing cancer in most cases. When signs and symptoms indicate the probability of tumor growth in a patient, a biopsy is performed to extract tissue samples from the patient by a pathologist. If the cells in the tumor tissues are identified as malignant, then genomic analysis of tumor DNA allows for the personalized treatments for cancer [10]. As cancers are genetic diseases [11] it is the outcome from the mutations in the cells. If the tumor is malignant then cells do not reproduce sexually [12] so, geneticist finds it even harder to track the links between tumors and genes by using classical inheritance method.

From the computational point of view, a number of graph-based approaches have been explored [13–15] to prioritize disease-gene associations. The major assumption of most of the graph-based approach is that gene causing the corresponding disease links to each other in a molecular network [16, 17]. The identification of new links or edges, i.e. new genetic interactions, is the most prominent task in biological network analysis. Most of the studies for gene-disease predictions make use of homogeneous networks, where all entities have the same type as well as the edges. In homogeneous networks, any shared neighbor between entities is considered the right approach for link predictions. However, in heterogeneous networks, the neighbors of an entity (or node) could have different types, and the number of shared neighbors can be a faulty parameter for predictions since it does not fully cover the graph’s heterogeneity. As a result, a different approach is required to predict novel links in a multi-layered network. Supervised machine learning has been used to predict gene-disease associations in heterogeneous networks. A popular approach makes use of the different relationship types in the multi-relational graph as a training feature for link prediction [18, 19]. In other gene-disease association studies from [20–22], the relations from the heterogeneous gene-disease graph have been exploited as features to prioritize the genes. One specific challenge in supervised link prediction is the need for training data, which has to be labeled for the two different types such as ***link*** and ***no link*** which is a time-consuming process.

Despite so much interesting research related to link prediction, all the above work avoid the impact of information diffusion mechanism on the link prediction. In the context of biological networks, the diffusion-based approach is considered important to identify genes and underlying diseases [23]. Classically, diffusion is studied mostly in a homogeneous networks [24, 25] where the information is diffused in single channels. Whereas, in the case of disease it propagates from different types of objects for e.g. disease propagate among genes and genes interact with each other. This process can be modeled as a 2-layered graph. In the context of our work, the first layer is tumor sample and gene association and second is the gene-gene interaction layer.

## Related work

In the context of general link prediction, matrix factorization is widely used. In matrix factorization, the networks are represented as matrix and entries are represented as the relationships. Menon et al.[26] claimed that link prediction is the problem of matrix completion. The low rank matrix decomposition based on Singular Value Decomposition (SVD) [27, 28] are used to predict links. For the multi-relational link prediction, tensor-based factorization is prominently used. The strength of tensors is that the multi-relational graph can be expressed in higher-order tensors which can be easily factorized. Unlike graphical models such as Markov Logic Networks (MLN) or Bayesian Networks, these models do not require a priori knowledge that needs to be inferred from data [29]. The matrix or tensor based factorization has three shortcomings. First, these methods do not account the structural property exhibited by networks such as high sparsity and skewed degree distribution [30]. Second, matrix or tensors based factorization methods requires the latent features or components to predict the links. It is difficult to estimate the number of latent features in advance that can give the best predictions. Third, if computational cost is an issue then matrix or tensor factorization can be very expensive and time-consuming [31, 32].

With the boom in neural network embedding, the problems of matrix factorization are overcome. Network embedding is the method to learn a low-dimensional representation of nodes in the network preserving network structure [33]. In recent studies, a node2vec [34] approach can analyze different network neighborhoods to embed nodes based on the assumption of homophily (i.e. network communities) as well as structural equivalence (i.e. structural roles of nodes) for link prediction in a homogeneous network for same the edge type. The study by Zitnik et al.[30] extended the node2vec algorithm in a multi-layered network called OhmNet, where each layer represents molecular interactions in different human tissues and reported accurate predictions for tissue cellular functions.

In recent years, the node embeddings techniques [30, 35] seems prominent because these methods have demonstrated high accuracy but it has also some limitations. These methods actually require learning steps which might be unfeasible for large-scale networks with millions of nodes [36]. Similarity-based propagation methods are also well studied in predicting the links in bipartite networks. This is the classic network based propagation in recommender system that predicts most relevant objects for users [37, 38].

In the biological context, diffusion-based approach for predicting disease and genes are well studied. Network propagation has become a demanding technique in computational systems biology with the focus on protein function prediction, disease gene prioritization, and patient stratification [39]. Similarly, network propagation approach are used to study the cluster-wide variety of cancer types [40, 41].

The study by Cowen et al.[23] reported that network-based propagation is a powerful data transformation method of broad utility in genetic research. Network propagation magnifies a biological signal based on the belief that genes underlying similar phenotypes influence to interact with one another [42]. The work by Ruffalo et al.[43] suggested that using network propagation can predict the cancer driver genes which tend to cluster in the network. There are different variants of network propagation proposed such as random walks [44], PageRank [45] and heat diffusion [46] algorithms. These methods are successfully applied to study biological problem. Among them heat diffusion algorithm showed potential in prioritizing the disease gene association [47, 48] and performs the best among all network-based diffusion approaches. Similarly, the HotNet algorithm [49] based on heat diffusion algorithm shows promising results to identify mutated genes. From the computational perspective, heat diffusion is fast to compute [39, 50, 51] and robust in memory usage [52].

The heat diffusion discussed above is different in two important ways. First, those heat diffusion-based approach is applied in a homogeneous network, meaning where nodes and the edges are of the same type. Second, we use 2 layered networks; the first is used for selecting seed nodes to carry tumor information and the second for diffusing the carried information in a genetic interaction network to predict the association between tumor samples and genes. Our approach is similar to DawnRank[53]. However, there are three key differences which are as follows. (i) Choice of the ranking algorithm which is heat diffusion instead of PageRank, (ii) Using information from multiple patients tumor sample data, (iii) Using methylated genes instead of the gene expression data.

## Methods

### Graph data model

We used 2 layered graph [54] to model our graph. The main motivation for using 2 layered graph is that using a single layer of Tumor and Gene graph information is not sufficient to model the complex process like tumor samples and gene link prediction. Tumors which are genetic disorders, it is important not to ignore the information about genetic interaction. Due to this, we need a separate layer to model genetic information. When gene interaction graphs are available, it is natural to incorporate them as additional information sources. This will improve the prediction accuracy of the model. The previous studies [55, 56] also showed that by the integration of multiple graphs can achieve higher accuracy than any single graph alone.

### Construction of network layer 1

**Fig. 1:**
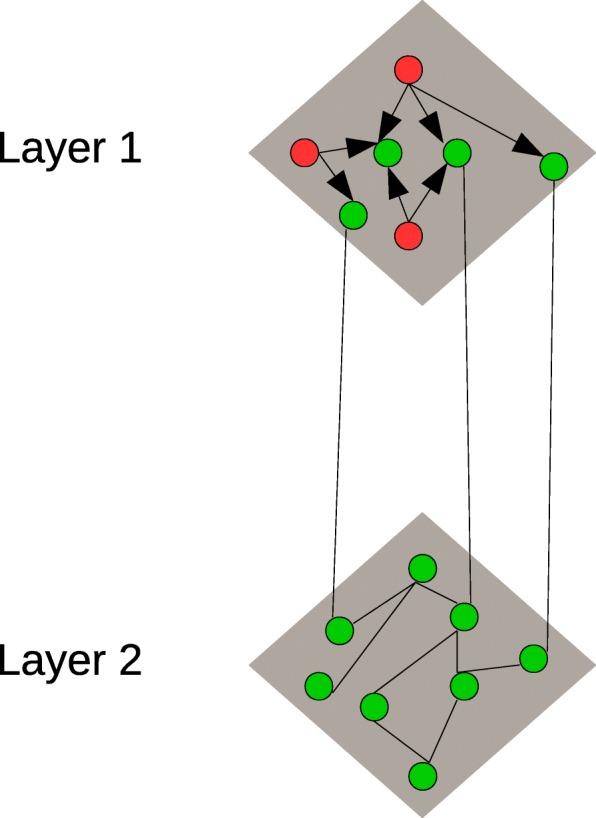
2-Layered Network of Tumor sample and Gene. Layer 1 is the bipartite graph of Tumor samples and Genes. Layer 2 is the undirected graph of Genes. Tumor sample is represented by red node and Gene is represented by green node in the figure. The Links between Tumor sample and Gene is directed link represented by directed arrow and the links between gene is undirected link represented by line

Figure 1 shows the first layer which is the bipartite graph between tumor samples and genes. This network is constructed from COSMIC (Catalogue Of Somatic Mutations In Cancer) Methylation RDF Data^1^. We use the COSMIC database because it uses the expert-curated information of somatic mutations in human cancers [57]. COSMIC has divided the datasets into logical categories, namely “Complete Mutation Data”, “Non-Coding Variants” and “DNA Methylation Data”. Our focus is on the “DNA Methylation Data" so we preprocessed the COSMIC RDF methylation data which has the properties: *id, sample name,location, gene names* and *methylation status*. Each *sample name* is a tumor sample of the patient and is extracted from different location of the body for example “TCGA-CV-A6JN-01" is a tumor sample and location is “Upper Aerodigestive Tract”.

**Fig. 2:**
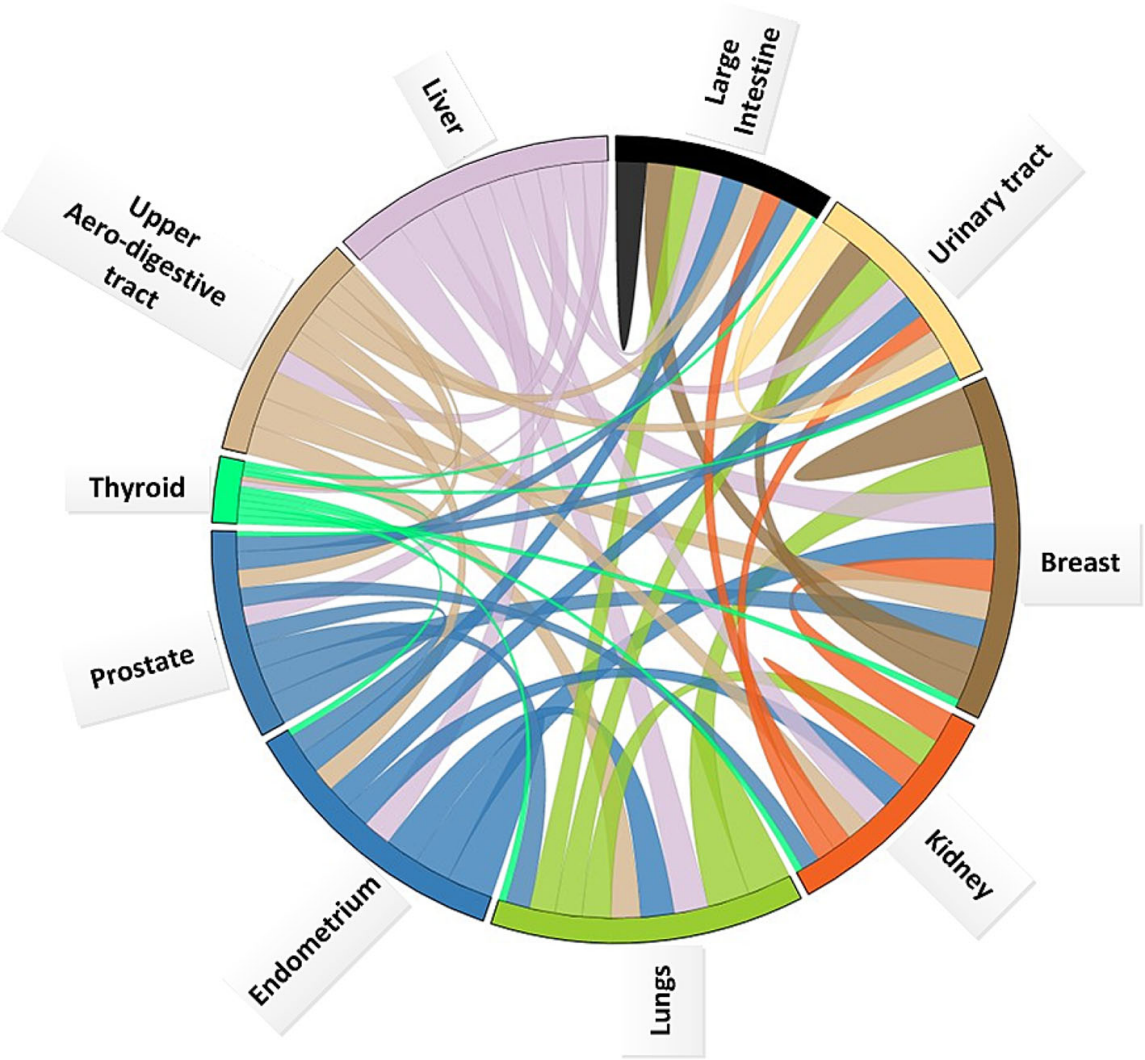
Visualization of the genes shared across the different anatomical location on the cosmic methylation data. The size of the ring means how many genes in the dataset are the members of the anatomical location of the body. An arc indicates how often this gene is shared across the connected segments

Figure 2 shows the gene across different anatomical location from COSMIC Methylation RDF data. The tumor samples in the datasets are taken from ten different anatomical locations. The *gene name* in the datasets is the accepted HGNC^2^ (HUGO Nomenclature Committee) identifier which provides the unique gene symbols and names for human loci.

The methylation level reported in the data is based on the beta value. The beta-value is the estimate of methylation level using the ratio of intensities between methylated and unmethylated genes. The genome location is based on the CpG targeted by the probe in the coding region. Our bipartite network consists of two disjoint sets of nodes: one set corresponds to the tumor samples; the other set corresponds to all the methylated genes in each tumor samples. The edge between the tumor sample and gene is based on the fact reported in the data. For instance, “TCGA-B6-A0RG-01” is a tumor sample and *“HOXC4”* is a methylated gene reported in the data then we link this relation by “hasGene” edge which is $\left [\text {TCGA-B6-A0RG-01}\ \underrightarrow {hasGene}\ \text {HOXC4}\right ]$.

### Construction of network layer 2

Network layer 2 is the interaction graph between genes as shown in Fig. 1. For the construction of network layer 2, we used 9 different variants of protein interaction channels. One from BioGRID and eight from STRING protein-protein interaction databases. These 2 databases are publicly available.

The prior studies by [47, 58, 59] modeled gene interaction graph as the undirected graph. We took the same approach and modeled our gene interaction graph as the undirected graph.

We used the protein-protein interaction links with weights for the **homosapiens** class from latest STRING version 10.5 database. There are eight different weighted channels of the protein-protein interaction networks available in STRING which are as follows: *co-expression, co-occurrence, database, experimental, fusion, neighborhood, textmining* and *combined*.

From BioGRID Api^3^ we constructed Gene-Gene physical network. BioGRID database provides the protein interactions curated from the biomedical literature [60] and has provided well validated *physical* interactions. The previous studies by [61–64] has shown that the potential of prioritizing the genes based on the physical properties. This network is the unweighted network.

To transform the original protein interaction network into a gene interaction network for both STRING and BioGRID data, we took the following approaches: (i) Protein names were mapped to their encoding genes by parsing of EnsEMBL files [65]. (ii) In the case of genes encoding multiple proteins, we took the edge of maximum (integrated) weight connecting any pair of proteins encoded by such genes. Similar technique for protein to gene mapping has also been used by the prior studies [48].

Table 1 shows the detailed summary of the nodes and edges used in the construction of the network.

**Table 1:** Network summary of 2-layered tumor-gene graph

Property	Value
Number of tumor samples	4086
Number of genes	4071
Number of relations between tumor samples and genes (hasGene)	222252
Co-occurrence	1166
Co-expression	208470
Database	23169
Experimental	170642
Fusion	98
Neighborhood	18929
Text mining	322883
Combined	358627
Physical	18395

### Solution approach

**Fig. 3:**
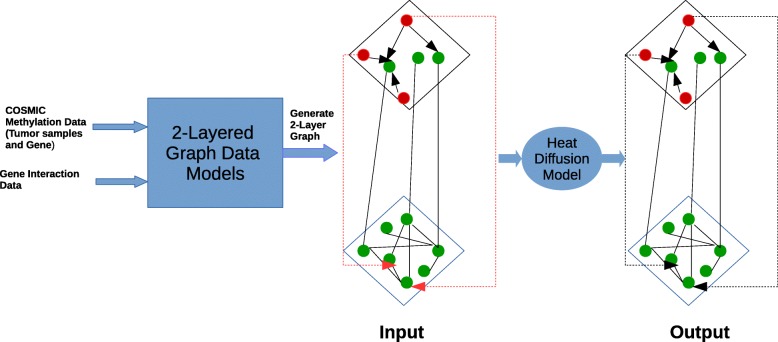
2 layered graph is constructed using 2 different data source (i) COSMIC methylation data for tumor samples and genes (ii) STRING or BIOGRRID database for genetic interaction. The input is 2-layered graph with missing links between tumor samples and genes. The heat diffusion is applied to the 2-layered graph containing missing links. The output is the final link prediction from the heat diffusion graph. Red node is a Tumor sample node and the green node is a Gene node. The Links between Tumor sample and gene node is represented by directed line whereas links between gene nodes is represented by undirected line. The red line is missing link and dotted black line a predicted link by heat diffusion algorithm

Our main aim is to predict the links between tumor sample and genes. For this, our first step is to use graph as an input. The input graph is the 2-layered network. This input graph has the missing links between the tumor samples and genes. In Fig. 3 shown by red arrows are the missing links that we want to predict.

Our second step is to apply the heat diffusion algorithm. For the execution of the heat diffusion algorithm, we need seed nodes. The seed nodes carry information about tumor samples. This information is available from the network layer 1 and diffused to gene-gene interaction layer. Once the diffusion process is over, then we get the association score between every tumor samples and genes. These association scores provide tumor samples and genes prediction.

### Model description

#### Heat diffusion model

Heat diffusion is the usual physical phenomenon. In a medium, heat always flows from a high temperature to a low temperature. The heat diffusion-based approaches have been successfully applied in various domains such as web spamming in web graph analysis [66], recommender system [67, 68] and disease-gene prioritization [47]. To make it self-contained, we will briefly introduce the heat diffusion model for weighted and unweighted undirected graphs which is adapted from Yang et al.[66].

#### Heat flow on an undirected and unweighted gene-gene interaction graph

We inferred the unweighted graph as the graph which has no edge weights. In the case of undirected and unweighted graph, the edge (*g*_*i*_,*g*_*j*_) is considered as a pipe from where the heat flows and connects gene nodes *g*_*i*_ and *g*_*j*_.

In an undirected graph, the heat can be modeled as follows. For instance, at time t, every gene node *g*_*i*_ obtains *M*(*i,j*,*t*,*Δt*) amount of heat from its neighbor gene node *g*_*j*_ for a time of *Δt*. We have two assumptions here:

- The heat obtained *M*(*i,j*,*t*,*Δt*) is proportional to the time period *Δt*.
- The heat obtained *M*(*i,j*,*t*,*Δt*) is proportional to the heat difference *f*_*j*_(*t*)−*f*_*i*_(*t*).
- *d*(*g*_*j*_) is the degree of the gene node *g*_*j*_.
- *f*(0) is the initial heat vectors of the genes.
- *f*(1) is the final heat vectors of the genes.

Furthermore, based on this assumption the amount of heat transfers between gene nodes is expressed as:

1 $$ \mathbf{f}(1)= e^{\alpha \mathbf{H}^{**}} \mathbf{f}(0)   $$

Where **H**^∗∗^ is the heat matrix for the undirected unweighted graph

2 $$ H^{**}_{ij}= \left\{\begin{array}{ll} -d\left(g_{j}\right), & \text{if}\ j=i \\ 1, & \left(g_{j},g{i}\right) \in E,\\ 0, &\text{otherwise,} \end{array}\right.  $$

#### Heat flow on an undirected and weighted gene-Gene interaction graph

In the case of the weighted links between the genes, we need to modify the heat diffusion model. Consider a weighted graph of genes such that *G*=(*V,E*,*W*) where V is the gene nodes such that *V*={*g*_1_,*g*_2_,*g*_3_,...,*gn*} On a weighted graph, in the pipe (*g*_*i*_,*g*_*j*_). *W* = *w*_*ij*_| gene *weight* score associated with edge (*g*_*i*_,*q*_*j*_). Suppose, at time t, each gene node *g*_*i*_ receives RH = RH(i, j, t; *Δ*) amount of heat from *g*_*j*_ during a period of *Δ*t. We made four assumptions as follows:

- RH is proportional to the time period *Δt*.
- RH should be proportional to the weight *w*_*ji*_ of the undirected edge (*g*_*j*_,*g*_*i*_).
- RH should be proportional to the heat at node *g*_*j*_.
- RH is zero if there is no link between *g*_*j*_ to *g*_*i*_. As a result, *g*_*i*_ will receive $\sum _{j:(g_{j},g_{i}) \in E} \sigma _{j}w_{ji}f_{j}(t)\Delta t$ amount of heat from its neighbors that are connected to it.
- $\sigma _{j} = \frac {\alpha }{d({g_{j}})}$ where *d*(*g*_*j*_) is the out degree of the gene node *g*_*j*_ and *α* is the thermal conductivity.

Simultaneously, node *g*_*j*_ diffuses DH(i,t, *Δ* t) amount of heat to its neighboring nodes. We consider that:

- The heat DH(i,t, *Δ* t) should be proportional to the time period *Δt*.
- The heat DH(i,t, *Δ* t) should be proportional to the heat at node *g*_*i*_.
- Each node has the same ability to diffuse the heat.
- The heat DH(i,t, *Δ* t) should be distributed to its neighboring nodes proportional to the weight on each edges.
- *τ* is the flag to check whether the node has any outgoing links. If there is any outgoing links then *τ*=1 else *τ*=0

Thus heat diffusion between gene nodes is given by,

3 $$ \mathbf{f}(1)= e^{\alpha \mathbf{H*}} \mathbf{f}(0),  $$

The **H**^∗^ which is the heat matrix for the undirected weighted graph is modeled as,

4 $$ H^{*}_{ij}= \left\{\begin{array}{ll} -(\frac{\tau_{i}}{d_{i}})\sum_{k:(i,k) \in E} w_{ik}, & \text{if}\ j=i \\ \frac{w_{ji}}{d_{j}}, & (g_{j},g{i}) \in E,\\ 0, &\text{otherwise,} \end{array}\right.   $$

The matrix $e^{\alpha H^{*}}$ is called as diffusion kernel which means the heat diffusion process continues infinitely many times from the initial heat diffusion. The parameter *α* is called as thermal conductivity. Higher the value of *α* faster is the spread of heat in the network. If *α* is infinitely large then heat diffuse from one node to another quickly.

In the context of gene-gene interaction, studies by [69–71] shown that the genes can randomly interact between adjacent genomic fragments. Thus, in order to capture that behavior, we add uniform random relations among different genes. Let *γ* denotes the probability of not forming random interactions and (1- *γ*) is the probability of taking a “random jump”. This behavior is also called “teleport” operation in the computation of PageRank [45] in web graph. The real world application considers the random edges [66] so, we followed the same setting of *γ* = 0.85 as in PageRank in all of our experiment.

Without any prior knowledge, we set $g = \frac {1}{n} 1$ where g is a uniform stochastic distribution vector, 1 is the vector of all ones, and n is the number of genes. We employed the above information and adapted our model as:

5 $$ f(1) = e^{\alpha R}f(0), R = \gamma H + (1-\gamma)g 1^{T}  $$

Where H can be replaced either *H*^∗^ or *H*^∗∗^ depending upon the kind of graph used.

#### Computational complexity

When the gene interaction graph is large, then the direct computations of *e*^*αR*^ is time-consuming so we adopted the discrete approximations by Yang et al.[66]:

6 $$ f(1) = \Bigg(I + \frac{\alpha}{M}R\Bigg)^{M} f(0),   $$

where *M* is the positive integer and *I* is the identity matrix. In order to reduce the computational complexity, we apply three methods: (1) Since *f*(0) is a vector, we iteratively calculate $(I + \frac {\alpha }{M}R)^{M} f(0)$ by applying the operator $(I + \frac {\alpha }{M}R)^{M} $ to f(0). (2) For matrix R, we apply a data structure which only stores information of non-zero entries, since it is a sparse matrix. (3) For every heat source which is tumor samples in our case, we bind it by diffusing heat to its neighbors. The selection of *α* and *M* parameters is detailed described in “Experiments” section. Specifically, after using the discrete formalization of the complexity of the heat diffusion algorithm in our model is given by *O*(*M*|*E*|*T*), where *M* is the number of iterations, *T* is the number of tumor nodes and |*E*| is the number of edges in the gene-gene interaction graph.

In the next section, we focus on how we use the diffusion model to predict Tumor samples and Gene relationships.

#### Tumor gene predictions in a toy network

**Fig. 4:**
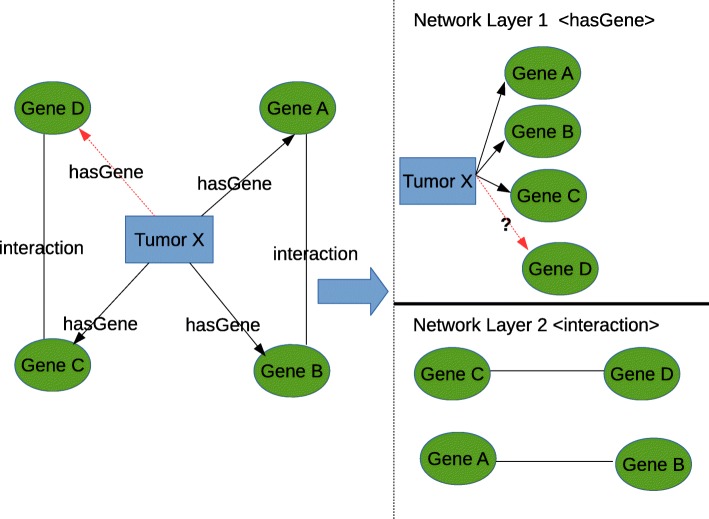
2-Layered Toy Networks of Tumor and Gene. The red arrow means the absence of link and the black arrow means presence of link

With the diffusion model described in the above section, we can now make the prediction by the following approach:

Let us consider a toy network as shown in Fig. 4. Network layer 1 is a Tumor sample and Gene layer and the network layer 2 is a gene-gene interaction layer. The initial temperature from **Tumor X** to **Gene A,B,C** and **D** in first layer at *t*=0 is given by the vector *f*(0):

The initial values of the vector *f*(0) is given by: *f*(0)=[ 1,1,1,0]^*T*^. We see in this vector the position of **Gene D** is 0 because there is no connection from **Tumor X** to **Gene D**.

The network layer 2 is a unweighted network so we model the heat matrix using the equation 2. Thus, our heat matrix is:

$$H = \left[\begin{array}{cccc} -1 & 1 & 0 & 0 \\ 0 & -1 & 0 & 0 \\ 0 & 0 & -1 & 1 \\ 0 & 0 & 1 & -1 \\ \end{array}\right] $$

Then heat diffusion at *t*=1 with *α*=1, is given by:

7 $$ f(1) = e^{\alpha H}f(0),  $$

Thus the computed *f*(1) vector is given by *f*(1)=[1.0,1.0,0.5,0.43]. Now normalizing the each vectors in *f*(1) by sum of all the numbers in *f*(1) then *f*(1) = [0.34,0.34,0.17, 0.14]. Here the interesting thing to observe is at the position of **Gene D**. This value was initially **0** after diffusion and normalization we saw the value to be **0.14**. This value is the likelihood of **Tumor X** to form link with **Gene D**.

## Experiments

From Eq. 6 we observed the two parameter *α* and *M*. We used the AUC-ROC evaluation metric which is commonly used in medical sciences and machine learning communities for quantifying the accuracy of prediction algorithm [72]. The brief description of this metric is provided in the “Evaluation metrics” section. The parameters alpha (*α*) and iterations (M) are estimated from 10-fold cross-validation on the training sets and applied the learned parameter in the test sets. Thus, AUC-ROC reported in the test set is the average score across ten folds.

***Impact of parameter*** *α*

Parameter *α* also known as thermal conductivity plays important role in heat diffusion process. If *α* is set to high then heat diffuses faster. Contrarily, heat diffuses slower. We varied *α* from the range 0 ≤*α*≤1. When *α* is set to **0**, that means no diffusion and the temperature distribution will remain exactly at the initial values than the structure of the graph.

**Fig. 5:**
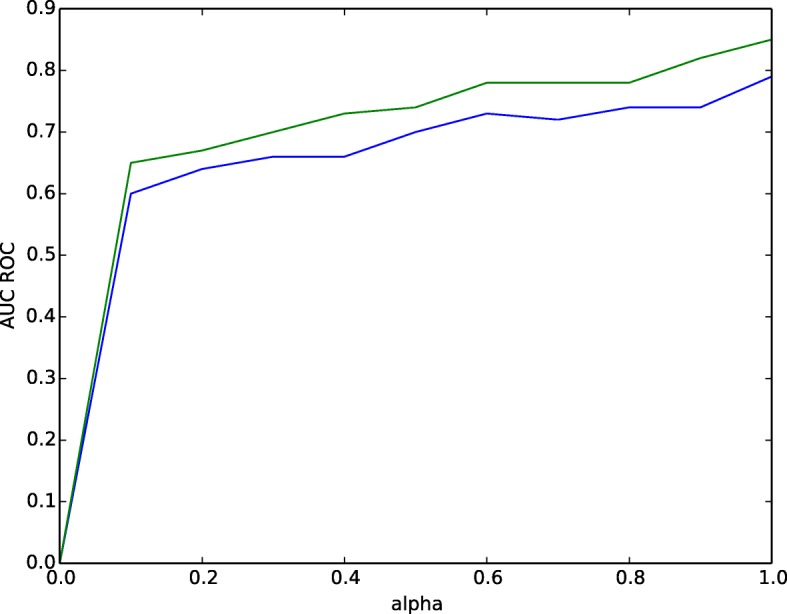
Impact of Parameter *α*. The prediction accuracy assessed by varying diffusion parameter ranging from 0 to 1 with the step of 0.1 in BioGRID and STRING Network to predict tumor samples and genes. Blue curve represents BioGRID and green curve represents String network respectively

To demonstrate impact of *α* in both **STRING** and **BioGRID** graph, we noticed the AUC-ROC score at different values of *α* in 25% testing set. As shown in the Fig. 5, we observed the increasing trend of AUC-ROC score with change in diffusion parameter *α*. The high AUC-ROC score of **0.74** for **BioGRID** and **0.85** for **STRING** in fusion channel is observed. After *α*≥1, there is no change in the AUC-ROC scores.

***Impact of parameter*** *M*

**Fig. 6:**
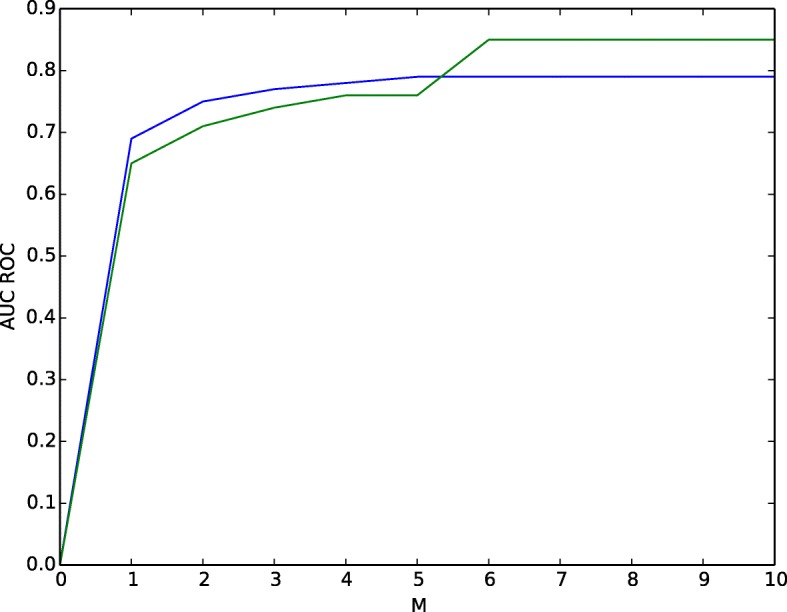
Impact of Parameter *M*. The prediction accuracy assessed by varying iterations in BioGRID and STRING Network to predict tumor samples and genes. Blue curve represents BioGRID and green curve represents String network respectively

The parameter *M* illustrates how distant the heat diffuses. From Fig. 6, we detected that when *M* = 5 for **BioGRID** and *M* = 6 for **STRING** graphs heat diffusion algorithm attains better performance in 25% testing set. After that, in both the graph the AUC-ROC score is constant. This means the heat diffusion algorithm is converged.

***Physical meaning of parameter****α* and m

The value for alpha and number of iteration M are computationally determined in the cross-validation mode in the training sets. These 2 parameters are quite hard to determine in advance without accessing any data. It has to be noted that the optimal performance obtained by maximizing alpha and M in our studies is based in terms of link prediction accuracy results. The mechanistic meaning of alpha is that if it approaches infinity the diffusion reaches to equilibrium then all the connected nodes receive the same diffusion contribution which is similar as PageRank. If we set alpha as 0 then there is no diffusion. Due to this, we need to find the optimum alpha that can balance the extent of heat which diffuses from genes to its immediate neighbors and to the rest of the network.

Similarly, M which is the number of iteration, the impact of this parameter is that how far the heat diffuses from the seed genes. The physical meaning of the scalar parameter M is the total time of diffusion, which controls the amount of heat to which the initial signal is allowed to spread over the network. The probabilistic interpretation for this computations is that if the input values are preference binary vector which is in our case (1 means genes having an association with tumor samples and 0 means no association) of starting positions for heat diffusing across the edges of the genetic interaction graph, the final value is the position distribution after M iterations. If M tends to infinity then the probability distribution approaches to a uniform distribution over all the genes.

***Runtime performance***

We performed the run time performance of the algorithm across all our gene-gene interaction graph. Table 2 shows the computational time for computing the link prediction between Tumor samples and Genes. From the study by [66] demonstrated that heat diffusion requires maximum of 30 iteration to converge. In a disease and gene prioritization setting, Nitsch *et al* [47] claimed that within 2 iterations heat diffusion gave reliable ranking of genes based on diseases.

**Table 2:** Runtime comparison on various gene-gene interaction channels

Data	Total run time		
	Iteration 2	Iteration 6	Iteration 30
Cooccurrence	29.05s	63.37s	242.24s
Experimental	27.01s	68s	267.60s
Fusion	45.65s	74.41s	278.78s
Neighborhood	25.62s	67.13s	260.46s
Textmining	29.55s	75.20s	264.93s
Coexpression	26.23s	65.97s	247.96s
Physical	25.46s	65.56s	234.31s
Combined	24s	61.15s	230s
Database	25.57s	57.28s	233s

From Fig. 6, we observed the impact of parameter *M*. At iteration = 6 the heat diffusion algorithm converged for Fusion channel in STRING data and for iteration = 5 for the physical channel in BioGRID data. For the rest of the channels, algorithm converged in less than 6 iterations. This means in maximum the algorithm takes **74.41** s to give us the prediction results for 4086 tumor samples and 4071 genes.

***Degree biasedness***

The heat diffusion process modeled using Eqs. 1 and 2 is biased towards the node having a high degree. The node having a high degree is influential because it is connected to many other nodes. Whereas Eqs. 3 and 4 is not biased towards the node having a high degree because each of the nodes is normalized by their degree. This makes every node to have the unit influence. To illustrate this, we ran our experiment in the setting of 75% train set and 25% test set to predict tumor samples and genes associations. The result is shown in Table 3.

**Table 3:** Impact of degree in gene-gene interaction channels

Data	Average node degree	AUC-ROC score without degree normalization	AUC-ROC score with degree normalization
Co-occurrence	6.08	0.50	0.84
Experimental	90.35	0.52	0.71
Fusion	1.66	0.80	0.84
Neighborhood	48.22	0.50	0.81
Textmining	159.4	0.52	0.75
Co-expresion	106.01	0.52	0.74
Physical	9.03	0.50	0.68
Combined	176.92	0.52	0.75
Database	20.19	0.50	0.75

From the results shown in the Table 3, we observed that after degree normalization the results are improved.

***Examples of tumor gene prediction results***

In total, we randomly selected 10 different tumor samples from our test set covering ten different tumor anatomical locations. Of the 8 interaction channels from STRING, we showed the result from fusion interaction channel because: (i) it gave us high mean AUC-ROC score compare to rest of channels (see “Results” section) (ii) as our study is related with tumor, gene fusions have been increasingly detected by next-generation sequencing (NGS) technologies based methods in malignant tumors [73,74].

We ranked the top 5 Genes predicted for each tumor sample based on their diffusion score.

From Table 4, we observed genes ***LAMA4, TNFRSF1A, IRS4*** and ***PCDH17*** are purely cancer genes^4^ which are predicted by the heat diffusion algorithm. The algorithm also identifies genes for a closely related anatomical location, for example, ***ZMAT4*** which is down-regulated for lung cancer. The algorithm predicts it for closely related anatomical location *upper aerodigestive tract*. Similarly, the ***SRI*** gene is considered as a useful marker of multi-drug resistance which may represent a therapeutic target for reversing tumor multidrug resistance [75]. This gene is top-ranked in both STRING and BioGRID by the algorithm. Furthermore, ***HOXC4*** is also ranked first in both BioGRID and STRING datasets by the algorithm for lung cancer. The studies by [76,77] also suggested the role of ***HOXC4*** involvement in lung cancer. *HOX* genes family are also known to behave as oncogenes for hematological malignancies and are often over-expressed in malignant cells [78].

**Table 4:** Example of tumor samples and gene predictions (*α* = 1.0)

Tumor sample name	Tumor location	alpha = 1.0 STRING (Top 5 Predicted Genes)	alpha = 1.0 BioGRID (Top 5 predicted genes)
TCGA-CV-A6JN-01	Upper aerodigestive tract	*RRBP1* *SPRY2* *ZMAT4* *HOXA2* *IRS4*	*HOXA2* *SPRY2* *IRS4* *ZMAT4* *RRBP1*
TCGA-SX-A71W-01	Kidney	*EPM2AIP1* *C4orf26* *TM4SF1* *DDAH2* *FBXO2*	*UBC* *APP* *PLVAP* *HNRNPA1* *ALB*
TCGA-38-7271-01	Lung	*HOXC4HOXA3HOXB3HOXB5* HOXD3	*HOXC4* *APP* *PRMT6* *WHSC1L1* *HOXB5*
TCGA-HP-A5MZ-01	Liver	*GNE* *GJC2* *LPPR2* *ACACB* *CLASP1*	*CLASP1* *LPPR2* *GNE* *GJC2* *ALB*
TCGA-D5-6541-01	Large intestine	*EDARADD* *AGRN* *ZMAT4* *MAGI2* *LAMA4*	*EDARADD* MAGI2 *LAMA4PCDHGC3ZMAT4*
TCGA-QU-A6IM-01	Prostrate	*SRI* *ZIC2* *ZIC5* *LPIN1* *IGF2BP1*	*SRI* *ZIC2* *NAGK* *POU5F1* *TEAD2*
TCGA-YC-A8S6-01	Urinary tract	*PRELID1* *SRM* *LSM4* *POLR2E* *CCT7*	*PRELID1* *CRYAB* *TNFRSF1A* *STOML2* *APP*
TCGA-A7-A5ZX-01	Breast	*C11orf53* *PCDH17* *KIFC2* *ZFR2* *PON3*	*PCDH17* *KIFC2* *ZFR2* *MAPK8IP2* *ILF2*
TCGA-EO-A3KW-01	Endometrium	*WT1-AS* *C20orf96* *NLRP10* *TP53I13* *OR4D6*	*PCDHGB1* *APP* *PCDHA10* *PCDHGA5* *HSPB2*
TCGA-DJ-A3VE-01	Thyroid	*MSLN* *ZMAT3* *TNFRSF1A* *SCNN1A* *MTA1*	*RDH5* *MSLN* *ZMAT3* *TNFRSF1A* *DUSP6*

The results shown in (Table 4) are randomly selected tumor samples from each anatomical locations and its ranked gene associations using fusion genetic interaction channel. We only showed the top 5 ranked genes examples for the particular tumor samples for the illustration purpose. This might have limited our results for not showing some of the potential and frequent cancer genes. So, we particularly took out the tumor samples for the breast and rerun our experiments this showed up the Tier 1 known cancer gene for breast such as *TP53, EGFR* and *BRCA1* in the top 100 list. The gene EGFR is shown up 15 times in the top 100 prediction list for breast tumor samples whereas *BRCA1* and *TP53* are shown up 2 times. The top 100 tumors and gene association showed the primary histology of breast carcinoma from cosmic methylation data. This result is shown in the Additional file 2: Table S2.

The heat diffusion algorithm has advantages for two reasons in our datasets (i) it can give us close link prediction between tumor sample and genes, (ii) it is faster to compute and robust in memory usage, which saves computational cost. [52].

## Results

In this section, we evaluate prediction accuracy of heat diffusion algorithm on STRING and BioGRID datasets.

### Evaluation metrics

We conducted the cross-validation by partitioning all tumor samples and gene relationships into ten folds and deleting the **hasGene** information of the tumor sample in the test set. We computed heat diffusion scores and ranked all tumor by their reconstructed tumor sample - gene relationship and recorded the Area Under the Receiver Operating Characteristics curve (AUC-ROC). The AUC-ROC metric can be understood as the probability that randomly chosen missing link is given a higher score than a randomly chosen nonexistent link [79].

To implement the AUC-ROC in the link prediction context, we took the following approach.

- The observed links E is randomly split into two parts: the training set *E*_*train*_ is treated as known information, while the test set *E*_*test*_ is used for testing and no information in the test set is allowed to be used for prediction. Thus total existing edge is then, *E*=*E*_*train*_∪*E*_*test*_ and *E*_*train*_∩*E*_*test*_=*ϕ*.
- Theoretically, this metric is computed as : *AUC*=(*n*^′^+0.5*n*^″^)/*n*. Where,
  1. n’: Number of time the missing links (links in *E*_*test*_) have a higher score than the non-existing links.
  2. n”: Number of times the scores of missing links is equal to a number of times the score of non-existing links (links in *U*−*E*), where *U* is the universal set.
  3. n: Number of independent comparison between missing and non-existing links.

This technique has been widely discussed in the link prediction literature [72,79*–*81]. If the AUC score exceeds 0.5 which means how better the algorithm performs than by pure chance. The proportion of the positives (links) and the negatives(no links) in our whole set data is 0.0133. This distribution is computed from the percentage of non zero entries from Tumor Samples and Gene Matrix from network layer 1.

**Fig. 7:**
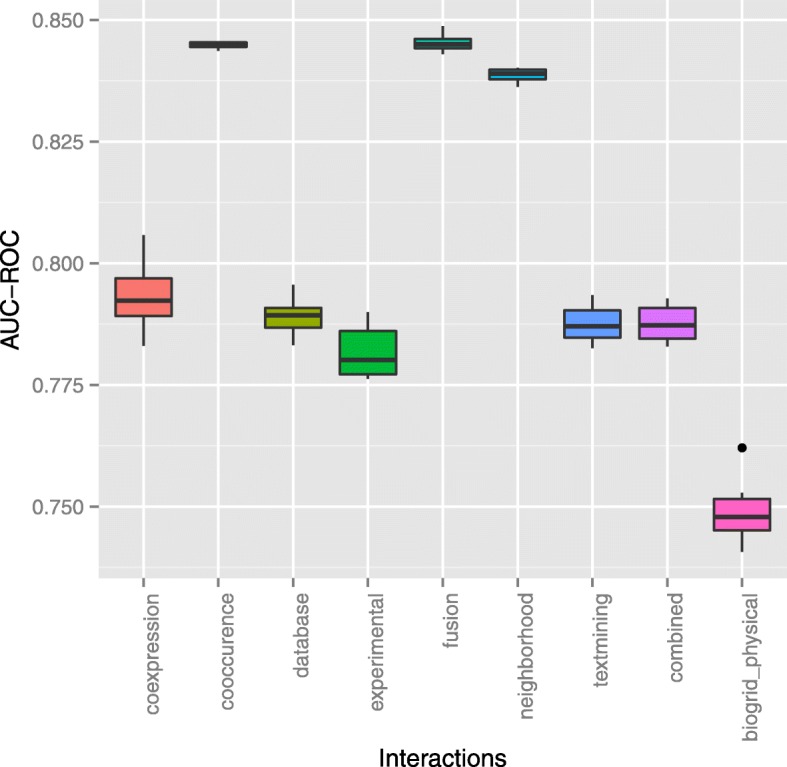
Result of 10 Fold Cross Validation in nine different gene interaction channels for predicting tumor samples and genes

The performance of the algorithm in 10-fold cross-validation across different channels is shown in Fig. 7. We observed that the mean AUC-ROC score is 0.84 for fusion and co-occurrence channel. Similarly, neighborhood channel has a mean AUC-ROC score of 0.83. These three channels do not have a significant difference in mean AUC-ROC scores. In STRING all three interactions aim to identify pairs of genes which appear to be under common selective pressures during evolution (more so than expected by chance), and which are therefore thought to be functionally associated [82]. A candidate gene fusion pair with a high score is more likely to be a driver gene fusion of tumor progression [74]. From here onward, we used fusion channel for the rest of our experiment.

The studies by [47*,*^83^*,*^84^] suggested that gene expression data potentially help in prioritize disease-gene associations. In Fig. 7, we observed mean AUC-ROC score of 0.79 using co-expression channel for predicting links between tumor sample and genes. The previous studies also suggested that [47*,*^83^*,*^84^] gene expression data potentially help in prioritizing disease-gene associations.

Consecutively, the other three genetic interaction approaches (i) textmining, (ii) database and (iii) combined channels each have mean AUC-ROC scores of 0.78. One reason for the combined channel to perform similar as textmining and database channel is that it contains scores of all the channels. As textmining approach might contain noise in the data which would have influenced the similar mean AUC-ROC score.

For BioGRID physical interaction channel without any weights between the gene pairs, the heat diffusion algorithm showed mean AUC-ROC scores of 0.74. This shows the potential of network propagation methods by only using network topology to predict the gene associations [23*,*^61^].

### Location based prediction

**Fig. 8:**
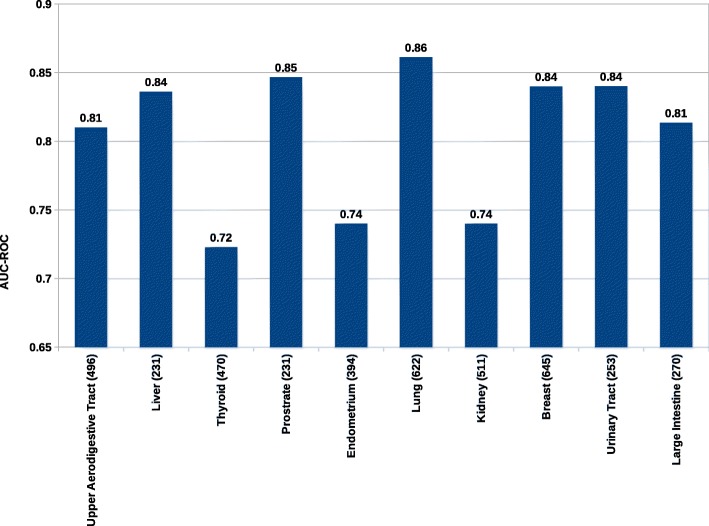
Area under ROC curve (AUC-ROC) scores for tumor samples and gene link prediction per location. Numbers in the brackets are counts of tumor sample for each anatomical location

To check the effectiveness of the heat diffusion algorithm, we tried to predict the genes for each anatomical locations using STRING fusion channel. The performance of the algorithm is shown in Fig. 8

This result is a demonstration of how tumor samples are related to gene predictions. We observed the highest AUC-ROC of 0.86 for predicting *lung* tumor samples and genes association. The lowest AUC-ROC of 0.72 for predicting *thyroid* tumor samples and gene associations. This means our computational network-based propagation and the data we used are not sufficient to explain the underlying mechanism of tumor gene associations in the thyroid section. Biologically, the study by [85*,*^86^] has also described the concept of aggressive clone and tumor heterogeneity in the case of thyroid tissue. So, this makes predicting the association between *thyroid* tumor samples and gene associations difficult.

### Comparison with the baseline algorithms

We compare our results from heat diffusion algorithms with baseline link prediction algorithm. To do this we applied several algorithms for link prediction such as scores based on similarity metrics namely Common Neighbors, Jaccard similarity, Adamic/Adar, Preferential Attachment and Resource Allocations. These algorithms are also called node based topological similarity algorithms because they can be viewed as computing a measure of ‘proximity" or “similarity" between nodes [87].

We also compared the results from heat diffusion algorithm with the two widely used path based similarity algorithms called Katz and Personalized PageRank algorithm. We used this algorithm in our 2-layered graph. From the first layer of Tumor-Gene graph we get the initial status of tumors for every genes. We used this status vector for every tumor samples and applied both algorithm.

- **Random Baseline** This simply assign each candidate edge a random score. This score is meant for the benchmark to compare other algorithms.
- **Node Based** The link prediction metrics assigns score of each candidate edges. These metrics presented by [87] are widely used in link prediction problem. However, using two different node sets tumor samples and genes cannot be directly applied in the context of bipartite graph because the neighbors of nodes on opposite sides of the network do not intersect. As the bipartite graph between tumor samples and genes is a directed graph we use the outgoing neighbors of tumor samples to the outgoing neighbors of the incoming neighbors of genes. If we consider tumors samples as *x* and genes as *y* then,
  The terms used in the Equation below can be described as:
  - *N*_*out*_(*x*) denotes outgoing neighbors of node x.
  - $N^{\prime }_{out,in}(y)$ can be interpreted as follows: (i) set of all the incoming neighbors of node *y*. (ii) From the list of neighbor of node *y* get all the list of outgoing neighbors.
  1. Common Neighbors: $score (x,y) = |N_{out}(x) \cap N^{\prime }_{out,in}(y)|$
  2. Jaccard’s Coefficient: *score*(*x,y*)=
$\frac {N_{out}(x) \cap N^{\prime }_{out,in}(y)|}{N_{out}(x) \cup N^{\prime }_{out,in}(y)|}$
  3. Adamic/Adar:
$score (x,y) = \sum _{z \in N_{out}(x) \cap N^{\prime }_{out,in}(y)} \frac {1}{log |N_{out} z|}$
  4. Preferential Attachment:
$score (x,y) = |N_{out}(x)| \cdot | N^{\prime }_{out,in}(y)|$
  5. Resource Allocation :
$score (x,y) = \sum _{z \in N_{out}(x) \cap N^{\prime }_{out,in}(y)} \frac {1}{|N_{out} z|}$
- **Path Based** Path based link prediction is based on the paths from one node to another. The two nodes are likely to be connected if there exist more paths between them. We employed the following metrics to compute the score between two sets of nodes:
  1. Katz: $score (x,y) = \sum _{i = 1}^{\infty } \beta ^{l} \cdot |paths_{x,y}^{< l>}|$
  2. Personalized PageRank: *score*(*x,y*) is explained as the probability of node *y* in a random walk that returns to node x with a probability *α* at each step, moving to a random neighbor with probability 1−*α*

Similarly, link prediction algorithm is also evaluated using AUC-PR metric. This metric is considered as more informative with heavy class imbalance problem such as link prediction [29*,*^88^*,*^89^]. This metric can perform robustly in a noisy environment [90]. Hence, in this study, we showed both AUC-ROC and AUC-PR evaluation metric for the link prediction by heat diffusion algorithm for the comparison with baseline.

The 10 Fold cross-validation result of the prediction result is shown in the Table 5:

**Table 5:** The result of 10 fold cross validation using heat diffusion algorithm with baseline prediction

Method	AUC-ROC	AUC-PR
Common neighbor approach (CN)	0.72	0.0382
Jaccard similarity (JS)	0.76	0.0549
Preferential attachment (PA)	0.73	0.0428
Resource allocation (RA)	0.78	0.0612
Adamic adar index (AAI)	0.79	0.0645
Katz (STRING) *α*=0.15,*β*=0.0001	0.54	0.0161
Katz (BioGRID) *α*=0.15,*β*=0.0001	0.72	0.0311
Personalized pageRank algorithm (PPR) (STRING)	0.81	0.0523
Personalized PageRank algorithm (PPR) (BioGRID)	0.81	0.0543
Heat diffusion algorithm (HD) (STRING)	0.85	0.0823
Heat diffusion algorithm (HD) (BioGRID)	0.74	0.0321
Random baseline (RB)	0.5	0.0125
Random network in layer 2 (RN)	0.5	0.0012

**Fig. 9:**
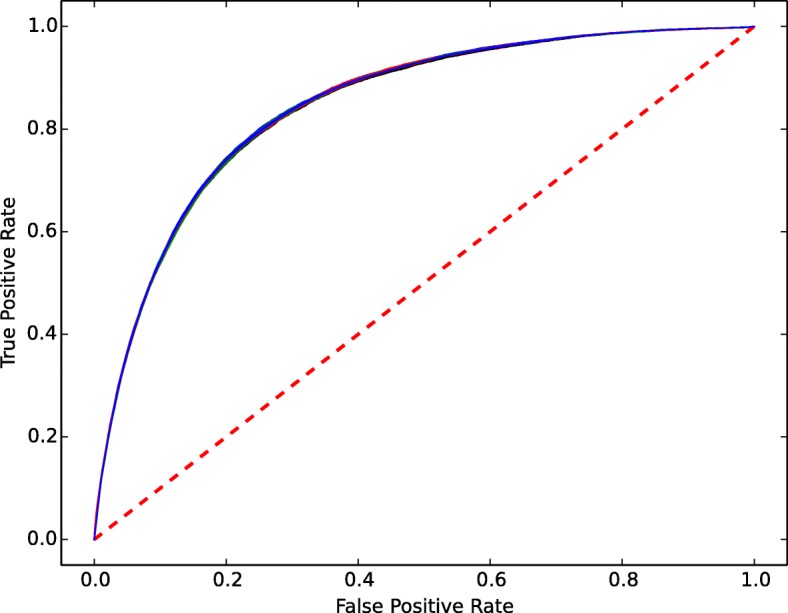
AUC-ROC curve plot. The red dotted line is the random guess. The Blue line represents the Mean AUC-ROC score from 10 fold cross validation

**Fig. 10:**
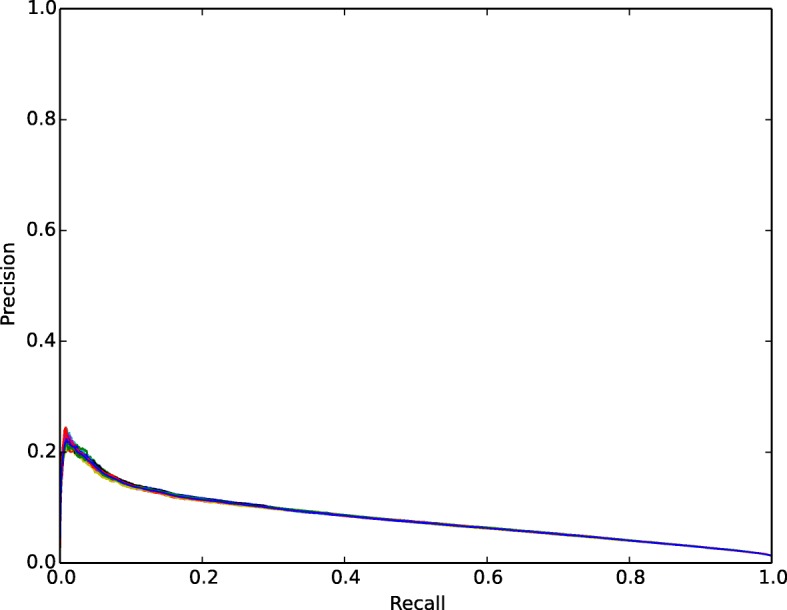
AUC-PR curve plot. The Blue line represents the Mean AUC-PR score from 10 fold cross validation

The heat diffusion algorithm outperformed other state of the art methods in predicting links between tumor samples and genes using STRING data. The AUC-PR and AUC-ROC curves are shown in Figs. 9 and 10. The standard deviation obtained from 10 fold cross-validation is very small and due to this the curves from the other folds superimposed with mean AUC-PR and AUC-ROC score and are not visible in the plots.

From Figs. 9 and 10, we see the disagreement between AUC-ROC and AUC-PR score in a link prediction task. AUC-PR curves consider the only prediction of the positives and are generally used for problems common in information retrieval, where negatives dominate the positives and are not considered important. For link prediction problem, AUC-PR curves give credit for correctly predicting edges but do not give credit for correctly predicting non-edges. This metric is heavily focused on predicting positives. This behavior harshly penalized for non-edges prediction. We believe this is one of the reasons for the discrepancies between the two metrics for the prediction performed by the algorithm. Whereas AUC-ROC is expected to be balanced metric for evaluating the accuracy of link prediction considering both edges and non-edges of the nodes. The prior study by [91] has also covered the anomalies of these discrepancies in the context of link prediction.

As different algorithms shared the same random trials. So, we applied the paired t-test to find out if there is a significant difference in the 10-fold cross-validation prediction results between heat diffusion and other state of the art methods at significant (*α*) level 0.05. The *p*-values of the test are reported in Table 6. We found that there is a significant difference between the prediction performed by heat diffusion with the state of the art algorithms. Though the heat diffusion methods outperform the Personalized PageRank method in both the AUC-ROC and AUC-PR performance metrics, the margin is not very high as shown in Table 5. The t-test shows the *p*-value at *α*=0.05 from the test is less than 2.2e-16 in both the case which suggests that there is a significant difference between AUC-ROC and AUC-PR score between these two methods. This holds true also in the context of the AUC-PR score by Personalized Page Rank Algorithm which is 0.05 and Heat Diffusion Algorithm which is 0.03 for the BioGRID datasets.

**Table 6:** The figure indicates the *p*-values of the t-test at significant level *α*=0.05, *** indicates highly significant

	CN	JS	PA	RA	AAI	Katz (STRING)	Katz (BioGRID)	PPR(STRING)	PPR(BioGRID)	RN (STRING)	RN (BioGRID)
HD (STRING)											
AUC-ROC	< 2.2e-16 ***	< 2.2e-16 ***	< 2.2e-16 ***	< 2.2e-16 ***	< 2.2e-16 ***	< 2.2e-16 ***	-	< 2.2e-16 ***	-	1.524e-14 ***	-
HD (STRING)											
AUC-PR	< 2.2e-16 ***	< 2.2e-16 ***	< 2.2e-16 ***	< 2.2e-16 ***	< 2.2e-16 ***	< 2.2e-16 ***	-	< 2.2e-16 ***	-	< 2.2e-16 ***	-
HD (BioGRID)											
AUC-ROC	< 2.2e-16 ***	< 2.2e-16 ***	< 2.2e-16 ***	8.611e-14 ***	< 2.2e-16 ***	-	2.667e-14 ***	-	< 2.2e-16 ***	-	8.839e-13***
HD (BioGRID)											
AUC-PR	< 2.2e-16 ***	< 2.2e-16 ***	< 2.2e-16 ***	< 2.2e-16 ***	< 2.2e-16 ***	-	0.00042 **	-	< 2.2e-16 ***	-	< 2.2e-16 ***

One of the important aspects of the heat diffusion is that it represents an exponential sum which converges more quickly in most cases than the geometric sum of Personalized PageRank [92]. This can be advantageous in the large graphs to get the desired results faster. In the biological context, similar work in prioritizing disease and genes [47] had already shown that the heat diffusion based ranking outperforms other diffusion methods in ranking disease-causing genes.

#### Comparison with the hotNet2 and hotNet heat diffusion algorithms

We compared the performance of our heat diffusion model with HotNet2 [93] and HotNet [94] algorithms. Both of the algorithms use heat diffusion model for the cancer genes network analysis. HotNet2 uses a directed heat diffusion model to determine the significance of mutations in individual genes and the local topology of interactions among the encoded proteins. Whereas, HotNet uses heat diffusion model to recognize the significantly altered subnetworks in the large genetic interaction network. We implemented both algorithms^5^ and apply in our methylation data to predict the links between the tumor samples and genes. The result is demonstrated in the Table 7:

**Table 7:** The result of 10 Fold cross validation using heat diffusion algorithm with HotNet and HotNet2 algorithms

Methods	Mean AUC-ROC score	Standard deviation
HotNet2	0.73	0.001889
HotNet	0.82	0.001835
Our heat diffusion method	0.84	0.001432

The major difference between HotNet2 and HotNet is how heat diffusion is modeled. The HotNet2 algorithm is modeled for a directed network whereas HotNet is modeled for the undirected network. As we implemented our heat diffusion model for the undirected network the result between HotNet and our approach is also similar. Though our approach has marginal improvement of the prediction accuracy. So, we performed paired t-test to check whether the difference is statistically significant between the 10 fold cross validation result by both methods. The *p*-value of the test (*p*-value = 4.489e-09) suggests there is the same problem in prediction performed by both the methods. The major technical difference in the HotNet and our heat diffusion model is how we propagate the heat. We used the discrete approximation of heat diffusion model $\left (I+\frac {\alpha }{M}R\right)^{M}$ [66] which has linear complexity whereas HotNet uses continuous diffusion kernel *e*^*αR*^ which has cubic complexity and for a huge graph this might be a problem. The linear kernel used in our approach is regarded as the random walk through the network which is comparable to the exponential or continuous diffusion kernel.

### Independent validation

In order to evaluate our heat diffusion model with independent datasets we chose STRING data because the heat diffusion model performed better in it in the cross-validation test. So, in the independent validation, we split our STRING data into 3 parts training (60%), validation(20%) and test sets(20%). The parameters for the diffusion are chosen from the training set. The model performed the AUC-ROC of 0.83 and an AUC-PR score of 0.078 in the test set.

### Statistical significance of the tumor-gene link prediction

**Fig. 11:**
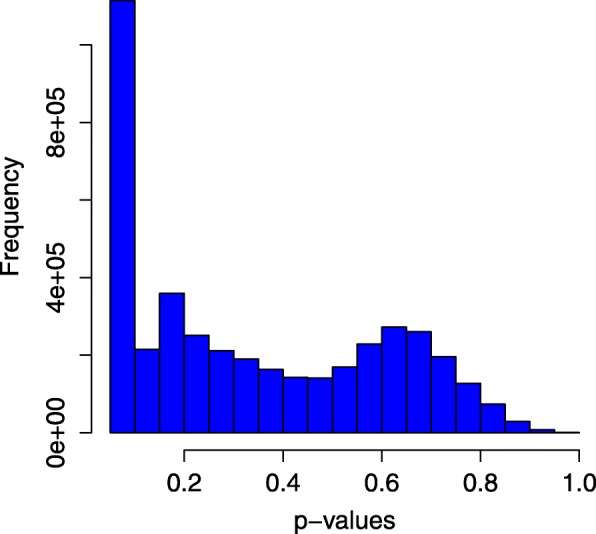
Histogram of *P*-values from Permutation test

To check the significance of the link prediction results, we performed the permutation test for predicted tumor sample and genes score. For this purpose, we partitioned the data randomly into training (75%) and testing (25%) sets. We recorded the heat diffusion scores in a test set using real gene-gene interaction data. After that, we randomize the gene-gene interaction graph by preserving the degree distribution and perform the heat diffusion process. The graph randomization process is repeated for 1000 times and *p*-values of the every tumor samples and gene prediction scores are computed as follows:

8 $$ \textit{p}-value(tumor,genes) = \frac{\Omega}{N}   $$

where *Ω* is the number of randomly produced tumor sample -gene links which receive higher heat scores than its actual predicted one. *N* is the total number of times the test is performed. The tumor samples and gene pair receiving higher *p*-values will be less likely to be an actual tumor-gene link because this pair will have a strong association with several randomly produced heat scores. The histogram of the *p*-values of our test is shown in Fig. 11.

We observed from the histogram that the large proportion of links are statistically significant (*p*-value are near to zero). However, for some links, the *p*-values are large, thus we have a risk of reporting false positives for a small proportion of tumor samples. This problem may be caused by the quality of the incomplete genetic interaction network from the STRING database.

As we have performed the test 1000 times, it is crucial to do the multiple hypothesis correction. The most common method to check this is to apply a Bonferroni correction test [95]. To do this we have to set up the new critical value which is $\alpha = \frac {\alpha *}{m}$. In our setting, *α*∗=0.05 and m = 1000, hence the new *α* of the individual test would be 0.05/1000 = 5e-05. Thus we consider those tumor-gene links with *p*-values <5e-05 to be significant. From the initial permutation test, we observed that there are 130913 tumor samples and gene links to be significant. Applying the Bonferroni correction criterion, with the *p*-values <5e-05 we found that only 62414 tumor samples and gene link to be significant.

### Biological meaning of the tumor sample and gene prediction

We examined the top 100 predictions performed by the heat diffusion algorithms. The results are ranked by heat diffusion scores. For each tumor sample and gene association, we looked at the biological meaning of the predicted links using the COSMIC database. This database collects somatic mutations from “The Cancer Genome Atlas (TCGA)" as well as from many smaller-scale studies and experimental studies [96]. Our results showed that the heat diffusion algorithm predicted 5 putative cancer gene *CDH10, CHST11, GRM3, VAV1* and *CCR4* from Tier 2 of the Cancer Gene Census^6^. Similarly 9 Tier 1 known cancer genes such as *TBX3, CNBP, CUX1, KLF6, HOXC13, FUS, BIRC3, GNAS* and *TNFAIP3*. These genes have documented evidence of their relevance to cancer. Not only the cancer genes but also heat diffusion identified 16 genes in which mutations are associated with altered drug sensitivity in cancer. The rest of the predicted tumor sample and genes have the evidence that the mouse insertional mutagenesis experiments support them as a cancer driver gene [97*,*^98^].

The results of the 100 predicted tumor samples and gene association with diffusion scores is provided in xlsx sheet as a Additional file 1: Table S1.

Out of the top 100 genes predicted, we found 9 tier 1 and 5 tier 2 cancer genes and 11 genes in which mutations are associated with altered drug sensitivity. The drug sensitivity information is identified by manually inspecting the COSMIC database. However, in a total of 4071 genes, we have a total of 244 (tier 1 and tier 2) cancer genes in our gene-gene interaction network. So we further investigated the statistical significance of the (14%) proportion of the top 100 predicted genes by randomizing gene-gene interaction graph using the Equation 8. The *p*-value of the test is 0.015 which suggest the 14 genes related to cancers out of top 100 predictions is statistically significant.

We further performed the test randomizing the network layer 1 which is the bipartite graph between tumor samples and genes by preserving the degree distribution. We found the nominal *p*-value to observe (14%) proportion of the top 100 predicted genes statistically significant (*p*-value <1e-3).

## Discussion

We considered two different baselines to compare the results of the algorithm. One is node-based and another is path-based algorithms. Out of the Node-based link prediction metrics, Adamic/Adar and Resource Allocation methods performed the best and Common Neighbor approach performed the worst in the datasets. In terms of AUC-ROC and AUC-PR, both Adamic/Adar and Resource Allocation have similar scores. Whereas, the heat diffusion algorithm has produced more accurate predictions, surpassing Adamic/Adar and Resource Allocation by up to 7.05% in STRING data with regard to AUC-ROC. While in the case of BioGRID data heat diffusion algorithm did not perform better in comparison with the Jaccard Similarity, Resource Allocation, and Adamic/Adar methods. Heat Diffusion algorithm performed better than Katz scores but worst in comparison with Personalized PageRank Algorithm for BioGRID datasets. One reason that heat diffusion performed better in STRING network in comparison to BioGRID is the coverage of the network. The network created from STRING is based on the assumption that it has integrated data from different sources which might affect the prediction results. Personalized PageRank algorithm surpassed all the link prediction methods for BioGRID datasets. In terms of AUC-ROC, Personalized PageRank algorithm gained 8% and in terms of AUC-PR 40% relative improvement over heat diffusion and Katz method for BioGRID datasets. The integration of gene interaction data in the diffusion model has proved to have a significant influence on the performance of the tumor samples and gene link prediction. One thing is also important to observe that only exploiting the link structures Personalized PageRank algorithm, outperforms several link prediction algorithms in both STRING and BioGRID datasets as shown in Table 5.

We observed heat diffusion algorithm outperform Personalized PageRank in STRING data. There is a gain of 4% in AUC-ROC scores using heat diffusion algorithms over Personalized PageRank algorithm which means the genetic interaction scores in heat diffusion are contributing to the improvement of prediction quality.

The heat diffusion algorithm also gives the nonexistent links which are not in the training set. In this work we did not further investigate about the nonexistent links because those evidences were not reported in the TCGA COSMIC database and we are unsure whether those are spurious or biological meaningful links. Though it is very relevant research direction to further investigate to find out the biological relevance of the nonexistent links predicted by the algorithm.

## Conclusion

We presented the heat diffusion algorithm, to predict links between tumor samples and gene in a 2-layer network. We used the heat diffusion algorithm to diffuse in 4086 independent tumor samples to 4071 genes. The heat is then diffused in nine different gene interaction channels. We noted that link prediction between tumor sample and genes gave us the highest AUC-ROC scores in fusion, co-occurrence and neighborhood channels in STRING data. The heat diffusion-based method gives us decent prediction even if no knowledge is available about the disease or phenotype and outperformed some of the baseline prediction such as Common Neighbors, Preferential Attachment and Katz methods. The other reason to choose heat diffusion is less memory intensive and faster to compute. In our experiment, we observed that the Personalized PageRank Algorithm also gave comparable results with heat diffusion methods. One of the advantages of using heat diffusion over Personalized PageRank method is that heat diffusion uses exponential sum, which converges quickly over personalized PageRank which uses geometric sum.

## Future work

There are several directions for future work. One of the important aspects would be the biological validation of the results, although we showed in our experiment that some of the top predictions per anatomical locations are indeed cancer genes. Computationally, we can evaluate the likelihood of identifying cancer genes if we run our algorithm in random data.

It is important to compare our results with other forms of somatic mutation data like copy number variation. In this work, we have not evaluated different cancer data for heat diffusion model. We only evaluated heat diffusion algorithms performance with a different state of the art link prediction algorithm for DNA methylation data. It would be important to see how heat diffusion algorithms perform in other somatic mutational data and compare against DNA methylation which could be a future work.

## Additional files

Additional file 1 The results of the top 100 predicted tumor samples and genes association with diffusion scores. (XLSX 11 kb)

Additional file 2 The resutls of the top 100 predicted tumor samples and genes association for breast carcinoma. (XLSX 7 kb)

## Data Availability

All the implementation of the algorithms is done in Python programming language. The statistical analysis is done using R. Supplementary data and source codes are available online at https://github.com/timilsinamohan/link_prediction_using_heat_diffusion. The whole cosmic methylation data used in this work can be downloaded from https://drive.google.com/drive/folders/1le0bWlasBb-AjKUbjjR1Ie16bxCAzv1D

## Footnotes

1
http://bioopenerproject.insight-centre.org/dataset

2
https://www.genenames.org/

3
https://wiki.thebiogrid.org/doku.php/biogridrest

4
http://ncg.kcl.ac.uk/cancer_genes.php

5
https://github.com/raphael-group/hotnet2/blob/master/hotnet2/network.py

6
https://cancer.sanger.ac.uk/census
